# Adjusting DBI-2016 to dietary balance index for Chinese maternal women and assessing the association between maternal dietary quality and postpartum weight retention: A longitudinal study

**DOI:** 10.1371/journal.pone.0237225

**Published:** 2020-08-20

**Authors:** Xiao Su, Wenli Zhu, Niuniu Li, Jing Sun, Yimin Zhu, Tan Liu, Haoye Xia, Zhiyong Dai, Yanchun Zhang, Lina Pan, Wei Jiang

**Affiliations:** 1 Department of Nutrition and Food Hygiene, National Health Commission Key Laboratory of Reproductive Health, Peking University School of Public Health, Beijing, China; 2 Peking-Ausnutria Maternal and Infant Nutrition Research Center, Beijing, China; Gazi University, Faculty of Health Sciences, TURKEY

## Abstract

Diet is believed to play a major role in maternal recovery, postpartum weight retention (PPWR) is one of the challenges for Chinese women. However, the association between puerperal women’s diet and PPWR remained unclear and complicated in China. The study assessed the dietary quality of puerperal women using adjusted Chinese Dietary Balance Index-16 (DBI-16) and explored its associations with PPWR. Participants were enrolled in the Mother-Infant Cohort Study of China. Dietary intake and demographic characteristics were obtained by a semi-quantitative food frequency questionnaire and a self-designed questionnaire at 0–3 months postpartum. PPWR was calculated by the weight difference at 0-3months and 6-8months postpartum minus pre-pregnancy weight. Dietary quality was assessed using adjusted DBI-16. 316 puerperal women were enrolled. According to adjusted DBI-16, 84.8% of participants had an insufficient dietary intake (vegetables 84.8%, fruits 91.8%, dairy 87.3%, soybean 61.4% and aquatic foods 79.4%, respectively), 67.1% had an excessive intake (cereals 60%, meat 57.3% and eggs 64.9%, respectively), 98.4% had an imbalanced diet consumption. PPWR at 0–3, and 6–8 months were 6.0 (±5.1) kg and 5.2 (±7.7) kg, and the percentage of PPWR (≥5kg) were 63.0% and 52.8% respectively. Multivariable linear regression showed the intake of fish and shrimp at 0–3 months postpartum was negatively associated with PPWR at 6–8 months (β = -0.114, SE = 0.279, *p* < 0.05). The diet quality of Chinese puerperal women was unreasonable and imbalanced. Fish intake tended to be a favorable factor for postpartum weight loss.

## Introduction

The dietary quality during the puerperium period is believed to play a major role not only in maternal recovery but also the breastfeeding infant’s development and health [1]. The Chinese Dietary Guidelines (2016) recommended a balanced and diverse dietary pattern for lactating women [2]. But the puerperal women always adhere to some Chinese traditional practices called “Zuo Yue Zi” or “doing the month”, including special diets and restricting outdoor activity, to restore balance and strength, compensate for blood loss, and increase breast milk production [3]. Puerperal women are prescribed special foods, including egg, animal viscera, pork feet, millet, chicken or carassius fish soup, noodles, various herbs, and brown sugar, inversely the fresh fruit and vegetables, cold foods and even seafood are always been restricted [4, 5]. Modern scientific guideline, traditional value and individual sociodemographic characteristics mix to yield influence on puerperal women’s diet, which remained unclear and complicated and needed to be focused on.

Given that dietary intake plays an important impact on maternal and offspring health, there have been growing interests in using dietary quality indices, such as Healthy Eating Index (HEI-2015) [6], Diet Quality Index (DQI) [7] and Mediterranean Diet Scale (MDS) [8], to evaluate the women’s adherence to a balanced diet. Components included in diet quality indices are usually nutrient adequacy, food diversity, and moderation [9]. In China, several dietary quality indices have been developed, such as Chinese Healthy Eating Index [10], Chinese Dietary Balance Index 2016 (DBI-16) [11]. The DBI-16 was developed based on Chinese dietary guidelines and Food Guide Pagoda (2016), to evaluate the overall quality, inadequacy, excess, and imbalance of diet by Lower Bound Score (LBS), Higher Bound Score (HBS) and Diet Quality Distance (DQD). Using the DBI assessment, the Chinese National Nutrition and Health Surveillance (2010–2013) showed that 73.6% of adults had moderate or severe inadequate intake of foods, 27.9% had a moderate or severe excess intake, and 7.0% had severe imbalanced diet consumption [12]. Because nutritional requirements are different according to age and sex, the general DBI for normal adults may not fit all target groups equally well, which has been revised/adjusted to meet the requirements of pregnant women [13], the elderly [14] and other specific populations except puerperal women.

Imbalanced diet could lead to many types of malnutrition [15], and pregnancy has been identified as a trigger for the development of obesity because of excessive weight gain [16] and long-term postpartum weight retention (PPWR) [17], especially the preferential accumulation of adipose tissue in the visceral compartment is an independent risk factor for a wide range of chronic diseases [18, 19]. The Chinese National Nutrition and Health Surveillance (2010–2013) showed the prevalence of PPWR (≥5 kg) in Chinese puerperal women was 41.5% [20]. Numerous studies have demonstrated the importance of diet quality on weight status in men and nonpregnant women, [21, 22] but very few studies have examined the association between individual dietary components and overall dietary quality with PPWR. On the other hand, an examination of overall dietary quality rather than individual nutrients or food intake can capture interactions between dietary components.

Thus, this study would revise the DBI-16 to meet the puerperal needs, to assess the overall dietary quality of puerperal women, and analyze its association with postpartum weight retention.

## Methods

### Participants

This study used data collected as part of the Mother-Infant Cohort Study (MICS) of China. The mother-infant pairs at 0–3 months postpartum were voluntarily recruited from June 2015 to December 2015, in seven cities (Beijing, Taiyuan of Shanxi, Jinan of Shandong, Changsha of Hunan, Shiyan of Hubei, Chongqing, and Chengdu of Sichuan) from northern, eastern, central and southwestern districts around China. The inclusion criteria of participant women in this study were as follows: being at 0–3 months postpartum, being apparently healthy without severe acute or chronic diseases, adopting breastfeeding. And the non-lactating women, whose infant did not receive breast milk during the previous day of investigation, were excluded. Based on this, the clinicians explained the study protocol to 540 mothers while their first-time postpartum visiting at local maternal and child care clinics. Finally, informed written consent was obtained from 316 participants voluntarily (the response rate was 58.5%). Even though there was not formula calculating, the sample size was sufficient to draw conclusion referring to literatures [19, 23–27].

The mother-infant cohort was followed up to 2 years postpartum until December 2018. During follow-up period, the dietary and feeding practices, physical and mental development of child, anthropometric indices of postpartum women and related factors were investigated. The women’s data at 0–3, 6–8 months postpartum were analyzed, including diet and weight data. The study was approved by the Peking University Institutional Review Board (Beijing, China, Approval Number IRB00001052-15020), and conducted according to the Declaration of Helsinki. The privacy of participant mothers and the confidentiality of their personal information would be protected.

### Measurements

#### Dietary intake

Maternal dietary assessment was carried out at 0–3 months by face-to-face interview with a 21-item food semi-quantitative Food Frequency Questionnaire (FFQ) adapted from the Chinese National Nutrition and Health Surveillance (2010–2013) and previously validated FFQ [28]. The original FFQ had 17 food groups, including 9 groups of fluids intake (different soups and drinks), that were thought important for maintaining lactation in Chinese traditional dietary culture. This study focused on the maternal health outcome (PPWR), so the original food groups were not fully suitable. On the other hand, the “Chinese Dietary Guideline” was revised in 2016, in which some foods were recommended specially for lactating women. Based on the above consideration, the original 17 food groups were modified to 21 groups in this study, namely rice, flour, coarse food grains, starchy roots, dark vegetables, light vegetables, fruit, livestock meat, poultry meat, eggs, aquatic products, marine fish, milk, legumes products, and soups, according to their nutritional significance, revised dietary guideline and puerperal women’s eating habits.

Women were asked to recall the quantity and frequency of food they consumed within one month postpartum (not the past one month) at the baseline (0-3m postpartum) or the 2nd (6-8m) follow-up survey. *Atlas of the Illustrative Food Pictures for Use in Dietary Intake Recall* were referred to help the participants to estimate the food portion size. The quantity (g) was reported according to frequencies of consumption (never, day, week, and month), and daily food intakes were calculated by multiplying the frequency of consumption for portion size (g).

#### Adjusting DBI-2016 to dietary balance index for puerperal women

General DBI-2016 was developed for normal adults according to *the Chinese dietary guidelines* and *Food Guide Pagoda (2016)*, which should be adjusted to meet the needs of puerperal women in the study. Revised DBI comprises five components, including (1) cereals; (2) vegetable and fruit; (3) bean and dairy; (4) animal food; (5) food variety. For each component and indicator, a score of ‘0’ indicates an excellent dietary intake, the negative score demonstrates that insufficient food intake and the positive score demonstrates that excessive food intake.

Negative score (range -12~0) is used to assess inadequate intake levels of vegetables and fruits, bean and dairy, which the guidelines recommend that puerperal women should consume in a “sufficient” or “plenty” amount. The score of the food variety component also ranges from -12 to 0. The “food variety” is assessed by intake of 12 identified food subgroups including: 1) rice and products; 2) wheat and products; 3) corn, coarse grains and products, Starchy roots and products; 4) dark-colored vegetables; 5) light-colored vegetables; 6) fruits; 7) soybean and products; 8) milk and dairy products; 9) livestock meat and products; 10) poultry and game; 11) eggs; 12) fish and shellfish. If the intake of the food subgroup reached or exceeded the lowest recommended intake scored 0, else, a score of -1 is assigned. The lowest recommended intake is 5g for soybean and products, and the other 11 food subgroup is 25g.

Moreover, both positive and negative scores were used to evaluate the intake levels of cereals ((range -12~+12) and animal food (range -12~-8), which the guidelines recommend consuming in an “appropriate” amount.

In considering the difference in nutrient requirements in energy consumption, the scoring of food components is based on seven energy intake levels from 1800kcal/d to 3000kcal/d. Scoring details of the revised DBI can be found in Table 1. Based on the scores for each DBI component, three indicators of diet quality are calculated. Higher Bound Score (HBS) is the sum of all the positive scores, which indicates excessive food intake. Lower Bound Score (LBS) is the sum of all negative scores, which indicates inadequate food intake; Diet Quality Distance (DQD) is computed by the absolute values of both positive and negative scores, which indicates imbalanced food intake. The lower and upper bound of HBS, LBS as well as DQD were: 0 to 20, 0 to 60, 0 to 60, respectively.

**Table 1:** Components of the adjusted dietary balance index scores for puerperal women.

Components	Score	Subgroup	Score	Intake range by energy levels based on the *Chinese Dietary Guidelines (2016)* (kcal/d)						
				1800	2000	2200	2400	2600	2800	3000
C1-Cereals	-12–12	Cereals ^a^	-12–12	<35g = -12;	<5g = -12;	<30g = -12	0g = -12;	<50g = -12;	<75g = -12;	<100g = -12;
				200–250g = 0;	225–275g = 0;	250–300g = 0	275–325g = 0;	325–375g = 0;	350–400g = 0;	375-425g = 0;
				>415g = 12.	>495g = 12.	>520g = 12	>600g = 12.	>650g = 12.	>675g = 12.	>700 = 12.
C2-Vegetables and fruits	-12–0	Vegetables	-6–0	≥ 400g = 0;	≥ 450g = 0;		≥ 500g = 0;			≥ 600g = 0;
				320–399g = -1;	360–449g = -1;		400–499 g = -1;			480–599g = -1;
				Score decreased by 1 with intake amount decreased by 80g; 0g = -6.	Score decreased by 1 with intake amount decreased by 90g; 0g = -6.		Score decreased by 1 with intake amount decreased by 100g; 0g = -6.			Score decreased by 1 with intake amount decreased by 120g; 0g = -6.
		Fruits	-6–0	≥200g = 0;	≥300g = 0;		≥350g = 0;		≥400g = 0;	
				160–199g = -1;	240-299g = -1;		280-349g = -1;		320-399g = -1;	
				Score decreased by 1 with intake amount decreased by 40g;	Score decreased by 1 with intake amount decreased by 60g;		Score decreased by 1 with intake amount decreased by 70g;		Score decreased by 1 with intake amount decreased by 80g;	
									0g = -6.	
					0g = -6.		0g = -6.			
				0g = -6.						
C3-Milk and dairy products, Soybean and soybean products	-12–0	Dairy	-6–0	≥400g = 0; Score decreased 1 with intake amount decreased by 80g; 0g = -6						
		Soybean	-6–0	≥15g = 0;		≥25g = 0;				
				Score decreased by 1 with intake amount decreased by 3g;		Score decreased by 1 with intake amount decreased by 5g;				
						0g = -6.				
				0g = -6.						
C4-Animal foods	-12–8	Meat and poultry products	-4–4	0g = -4;		0g = -4;			0g = -4;	
				1–20g = -3;		1-25g = -3;			1-35g = -3;	
				21–40g = -2;		26-50g = -2;			36-70g = -2;	
				41-50g = -1;		51-75g = -1;			71-100g = -1;	
				51-70g = 0;		76-95g = 0;			101-120g = 0;	
				71-90g = 1;		96-120g = 1;			121-155g = 1;	
				91–110g = 2;		121-145g = 2;			156-190g = 2;	
				111-130g = 3;		146-170g = 3;			191-225g = 3;	
				>130g = 4.		>170g = 4.			>225g = 4.	
		Fish and shrimp	-4–0	0g = -4;		0g = -4;			0g = -4;	0g = -4;
				1-20g = -3;		1-30g = -3;			1-35g = -3;	1-45g = -3;
				21-40g = -2;		31-60g = -2;			36-70g = -2;	46-90g = -2;
				41-60g = -1;		61-85g = -1;			71-110g = -1;	91–135 = -1;
				>60g = 0.		>85g = 0.			>110g = 0.	>135g = 0.
		Eggs	-4–4	0g = -4;	0g = -4;					
				1-10g = -3;	1-15g = -3;					
				11-20g = -2;	16-30g = -2;					
				21-30g = -1;	31-45g = -1;					
				31-50g = 0;	46-55g = 0;					
				51-60g = 1;	56-70g = 1;					
				61-70g = 2;	71-85g = 2;					
				71-80g = -3;	85-100g = 3;					
				>80g = 4.	>100g = 4.					
C5-Food variety	-12–0	Food variety ^b^	-12–0	≥12 kinds of food (soybean is 5g) = 0; Score decreased by 1 with food variety decreased by 1.						

^a^ Cereal include rice, wheat, legumes (except soybean) and tubers. Sweat potato: intake amount divided by 3; potato: intake amount divided by 4; yam and yambean: divided by 6; Score increased (decreased) by 1 with intake increased (decreased) by 15g while energy level of 1800 kcal, 20g of 2000 and 2200 kcal, and 25g of more than 2400 kcal;

^**b**^ Food variety: 1) Rice and products; 2) Wheat and products; 3) Corn, coarse grains and products, Starchy roots and products; 4) Dark-colored vegetables; 5) Light-colored vegetables; 6) Fruits: Fresh and dried fruit; 7) Soybean and products,: Soybean, black bean, bean curd; 8) Dairy products; 9) Livestock meat products; 10) Poultry products; 11) Eggs; 12) Fish and shellfish.

Each indicator is further divided into five levels according to scores: 1) Score “0” represents “excellent” dietary intake; 2) Less than 20% of total score represents “good” dietary intake; 3) 20%~40% of total score represents “mild poor” dietary intake; 4) 40%~60% of total score represents “moderate poor” dietary intake; 5) Higher than 60% of total score represents “severe poor” dietary intake.

#### Postpartum weight retention

Maternal self-reported and hospital-documented gestational weight gain (GWG) at 0.5~2.5 years after childbearing had been observed to be highly correlated [29]. Thus, in our study, body weight was reported by puerperal women themselves. The postpartum weight retention (PPWR) was calculated by subtracting the pre-pregnancy body weight from postpartum body weight at 0–3, 6–8 months postpartum. And gestational weight gain equaled to the weight before delivery minus the pre-pregnancy weight.

#### Related factors

We used a self-designed questionnaire to collect the sociodemographic characteristics (age, family affluent level, education level), gestational weight gain, delivery mode, feeding patterns. Education level included two levels: primary/secondary (high school or below), tertiary (bachelor degree or above). Feeding patterns were classified as follow: 1) Exclusive breastfeeding (breast milk was exclusively given without a very small amount of water or/and juice); 2) Predominant breastfeeding (breast milk was predominantly given with a very small amount of water or/and juice); 3) Mixed feeding (a mixture of breast milk and formula milk was given with or without some water or/an juice) [30]. Body mass index (BMI) was computed as weight in kilograms divided by the square of the height in meters (kg/m^2^). The BMI cut-off points for Chinese adults were used to determine underweight (BMI < 18.5 kg/m^2^), normal weight (BMI 18.5–23.9 kg/m^2^) and overweight (BMI 24.0–27.9 kg/m^2^)and obesity(BMI ≥ 28.0 kg/m^2^) [31]. Household monthly income per capita reported in CHY (China Yuan, 1CNY = 0.14 USD) and classified into 3 categories: low (less than 3000 CNY), middle (3001–6000 CNY) and high (above 6000 CNY).

### Statistical analysis

Data were input to EpiData3.1 software. All analyses were performed with SPSS software version 22.0 (SPSS Inc., Chicago, IL, USA). Numeric variables that normally distributed were given as mean ± standard deviation (SD), Numeric variables that non-normally distributed were given as median. Categorical variables were given as a percentage (%). Kruskal-Wallis test and Mann-Whitney U test were used to compare the distribution of HBS, LBS, DQD among socio-demographics and related factors.

Multiple linear regression analysis was utilized to evaluate the association between DBI scores for each component (cereals, vegetables, fruits, dairy, soybean, meat and poultry products, fish and shrimp, eggs, as well as food variety) and postpartum weight retention at 6–8 months postpartum. And the covariates included demographic characteristics (age, education level, and family income), delivery mode, infant feeding pattern, pre-pregnancy BMI, and gestational weight gain, some of which were valued as below: 1)Education level: high school or below = 0, bachelor degree or above = 1; 2)Family income (CNY): less than 3000 = 0, 3001~6000 = 1, more than 6000 = 2; 3)Delivery mode: vaginal = 0, cesarean = 1; 4)Infant feeding pattern: exclusive breastfeeding = 0, predominant breastfeeding = 1, mixed feeding = 2; 5)Number of childbirth: more than one child = 0, one child = 1. Multi-collinearity among covariates was estimated though tolerance, which being greater than 0.1 indicates that there is no multicollinearity among covariates.

The level of significance was p values<0.05 (two sides significance level).

## Result

### Characteristics of the study puerperal women

Characteristics of 316 puerperal women are presented in Table 2. The age range was 20–45 years and 83.5% of the participants were less than 35 years old. Most of the puerperal women (60.9%) had a bachelor degree or above as well as 71.4% had a middle or above household income (>3000 CNY). The majority (65.8%) was the first-time mother and 57.3% of the puerperal women adopted exclusive or predominant breastfeeding. The sample covered a wide range of pre-pregnancy BMI, but most of the participants (68.4%) had a normal weight, as well as 14.6% were overweight or obesity. Average gestational weight gain was 14.6 ±5.1kg. Postpartum weight retention at 0–3, and 6–8 months were 6.0 (±5.1) kg and 5.2 (±7.7) kg respectively, and percentage of PPWR (≥5kg) was 63.0% and 52.8%.

**Table 2:** Demographic characteristics of the participants (N = 316).

Characteristics	n	Percentage (%)
Age (years)		
Mean±SD	30.5±4.1	
20–34	264	83.5
35–45	52	16.5
Education level		
High school or below	120	39.1
Bachelor degree or above	187	60.9
Household monthly income per capita (CNY)		
≤ 3000	83	28.6
3001–6000	118	40.7
> 6000	89	30.7
Number of childbirth		
1	208	65.8
≥2	108	34.2
Delivery mode		
Vaginal	180	58.3
Cesarean	129	41.7
Feeding pattern at 0–3 months		
Exclusive breastfeeding	70	22.2
Predominant breastfeeding	111	35.1
Mixed feeding	135	42.7
Pre-pregnancy BMI (kg/m^2^)		
Mean±SD	21.2±3.1	
Underweight (< 18.5)	54	17.1
Normal (18.5–23.9)	216	68.4
Overweight (24.0–27.9)	34	10.8
Obese (≥28.0)	12	3.8
Gestational weight gain (kg, Mean±SD)	14.6±5.1	
Postpartum weight retention		
0–3 months		
Mean±SD	6.0±5.1	
<5kg	117	37.0
≥5kg	199	63.0
6–8 months		
Mean±SD	5.2±7.7	
<5kg	149	47.2
≥5kg	167	52.8

BMI: Body mass index

CNY: China Yuan, 1CNY = 0.14 USD

SD: standard deviation

### Assessment of food intakes by DBI components in puerperal women

Table 3 shows the scores for the DBI components and the percentage of participants with each score (estimated energy intake of 2400kcal per day). Overall the percentage of participants meeting the recommended dietary intakes was ranged from 8.9% to 38.6% (score = 0). A total of 60.0%, 57.3% and 64.9% of the puerperal women had excessive intakes (score>0) of cereals, meat and eggs, and 84.8%, 91.8%, 87.3%, 61.4% and 79.4% of the women had inadequate intake (score<0) of vegetable, fruits, dairy, soybean and fish. Almost 92.7% of the participants hadn’t achieved enough food variety.

**Table 3:** Scores for the DBI components and the percentage of the participants with each score (%) ^a^.

Score	Cereals	Vegetables	Fruits	Dairy	Soybean	Meat and poultry	Fish and shrimp	Eggs	Food variety
(-12) ~ (-11)	0.3	—	—	—	—	—	—	—	0.0
(-10) ~ (-9)	0.6	—	—	—	—	—	—	—	0.9
(-8) ~ (-7)	3.5	—	—	—	—	—	—	—	6.4
(-6) ~ (-5)	7.0	27.2	45.3	45.5	36.5	—	—	—	16.1
(-4) ~ (-3)	9.2	40.2	44.3	28.2	16.4	13.6	60.1	4.4	33.9
(-2) ~ (-1)	10.5	17.4	2.2	13.6	8.5	21.8	19.3	11.1	35.4
0	8.9	15.2	8.2	12.7	38.6	7.3	20.6	19.6	7.3
1~2	8.8	—	—	—	—	15.5	—	7.5	—
3~4	8.9	—	—	—	—	41.8	—	57.4	—
5~6	7.6	—	—	—	—	—	—	—	—
7~8	4.7	—	—	—	—	—	—	—	—
9~10	4.1	—	—	—	—	—	—	—	—
11~12	25.9	—	—	—	—	—	—	—	—
Total	100.0	100.0	100.0	100.0	100.0	100.0	100.0	100.0	100.0

^a^: The score range was different for each food subgroup: vegetables (-6~0), fruits (-6~0), dairy (-6~0), soybean (-6~0), meat and poultry (-4~4), fish and shrimp (-4~0), eggs (-4~4), food variety (-12~0).

### Distribution of overall diet quality of puerperal women by adjusted DBI

**Fig 1:**
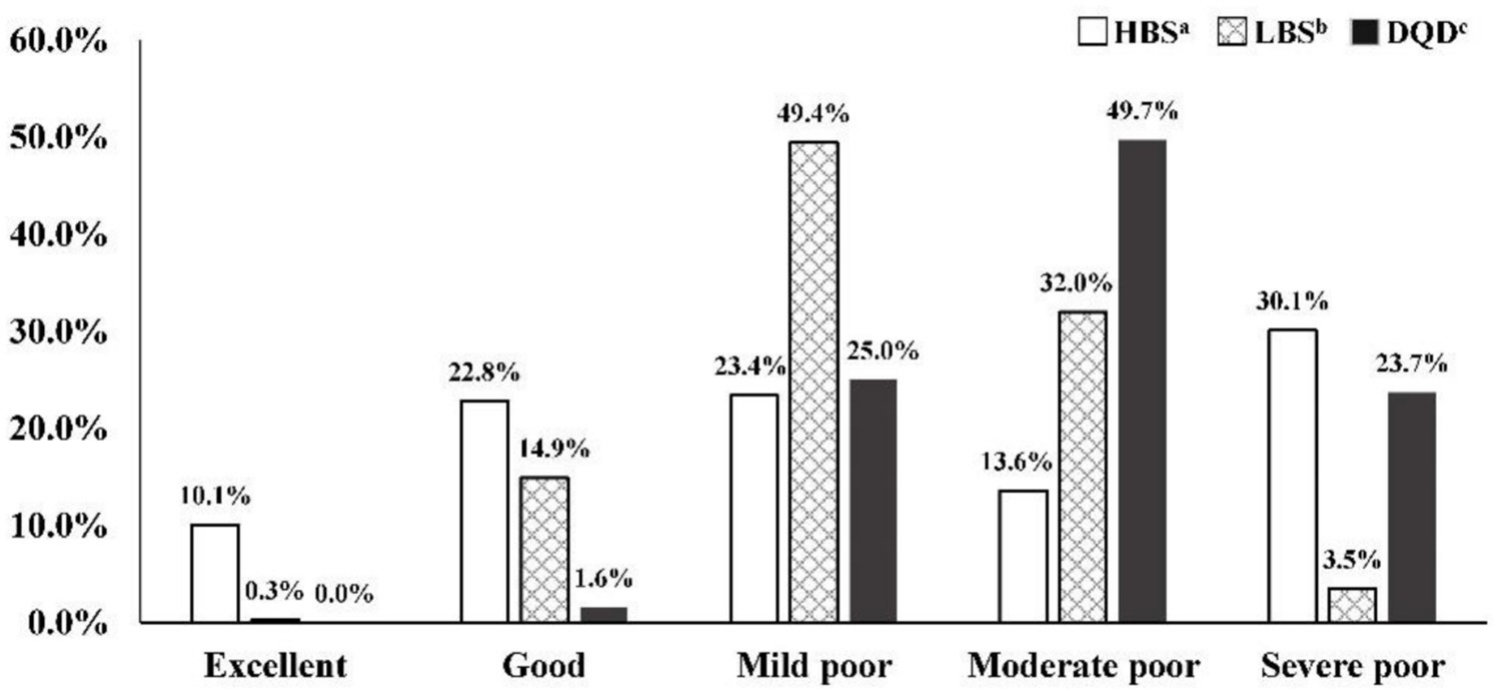
Distribution of DBI indicators among puerperal women. ^a^ HBS: Higher Bound Score of Dietary Balance Index for urban Chinese lactating women, excessive intake indictor.; ^b^ LBS: Lower Bound Score of Dietary Balance Index for urban Chinese lactating women, inadequate intake indicator; ^c^ DQD: Diet Quality Distance of Dietary Balance Index for urban Chinese lactating women, overall imbalanced food intake indictor. Score range of HBS is 0–20; No problem: 0; Almost no problem: 1–4; Low level:5–8; Moderate level: 9–12; High level: 13–20; Score range of LBS is 0–60; No problem: 0; Almost no problem: 1–12; Low level: 13–24; Moderate level: 25–36; High level: 37–60; Score range of DQD is 0–60; No problem: 0; Almost no problem: 1–12; Low level: 13–24; Moderate level: 25–36; High level: 37–60.

The median of overall HBS, LBS, and DQD were 7.5 (0–20), 22 (0–49), and 30 (10–54), respectively. Fig 1 presents the distribution of these indicators. The sum of the proportions of HBS, LBS and DQD in “excellent” and “good” level were 32.9%, 15.2%, 1.6% respectively. The sum of the proportions of HBS, LBS and DQD in the poor level were 67.1%, 84.8%, and 98.4%, respectively. The results showed that more than two-thirds of puerperal women had an excessive dietary intake, more than four-fifths of them have an inadequate dietary intake, and most of them have an imbalanced diet.

According to sociodemographic characteristics, there were no statistically differences for HBS, LBS and DQD (*p*>0.05), as shown in Table 4

**Table 4:** Distribution of DBI indicators according to characteristics (median).

Characteristics	HBS		LBS		DQD	
	Median	*p* value	Median	*p* value	Median	*p* value
Age groups (years)						
20–34	8.0	0.426	22.0	0.495	30.0	0.693
35–45	7.0		22.0		30.5	
Education level						
High school or below	7.0	0.557	22.0	0.595	30.0	0.879
Bachelor degree or above	8.0		22.0		30.0	
Household monthly income per capita (CNY)						
≤ 3000	10.0	0.250	23.0	0.210	31.0	0.097
3001–6000	6.0		22.0		29.0	
> 6000	8.0		21.0		30.0	
Number of childbirth						
1	8.0	0.066	22.0	0.751	31.0	0.053
≥2	7.0		22.0		29.0	
Delivery mode						
Vaginal	8.0	0.520	22.0	0.496	30.0	0.811
Cesarean	7.0		22.0		30.0	
Feeding pattern						
Exclusive breastfeeding	8.0	0.796	22.5	0.500	31.0	0.615
Predominant breastfeeding	8.0		21.0		29.0	
Mixed feeding	7.0		22.0		30.0	
Pre-pregnancy BMI (kg/m^2^)						
Underweight	9.0	0.353	23.5	0.127	32.0	0.233
Normal	7.0		21.0		30.0	
Overweight/Obese	5.0		24.0		31.0	

DBI: Chinese diet balance index; HBS: higher bound score; LBS: lower bound score; DQD: diet quality distance

### Association of dietary quality and PPWR

Multivariable linear regression analysis showed after adjustment for age, education level, family income, delivery mode and breastfeeding covariates, postpartum weight retention at 6–8 months was negatively associated with pre-pregnancy BMI (*β* = -0.302, *p* < 0.05) and fish and shrimp intake score (*β* = -0.114, *p* < 0.05), as well as positively correlated with feeding pattern (*β* = 0.135, *p* < 0.05) and gestational weight gain (*β* = 0.124, *p* < 0.05). Postpartum weight change between 6–8 months and 0–3 months was negatively correlated with gestational weight gain (*β* = -0.322, *p* < 0.05), pre-pregnancy BMI (*β* = -0.261, *p* < 0.05), and number of childbirth (*β* = -0.118, *p* < 0.05) as well as positively correlated with feeding pattern (*β* = 0.130, *p* < 0.05), as shown in Table 5.

**Table 5:** Multivariable linear regression model for postpartum weight retention.

Independent variables	PPWR at 6–8 months			Postpartum weight change (*PPWR*_*6-8month*_*−PPWR*_*0-3month*_)		
	*β*	*SE*	*p*	*β*	*SE*	*p*
Pre-pregnancy BMI	-0.302	0.150	*<*0.001^a^	-0.261	0.149	*<*0.001^a^
Gestational weight gain	0.124	0.084	0.031^a^	-0.322	0.083	*<*0.001^a^
Infant feeding pattern	0.135	0.538	0.016^a^	0.130	0.535	0.022^a^
Score of fish and shrimp intake	-0.114	0.279	0.044^a^			
Number of childbirth				-0.118	0.892	0.040^a^
R^2^	0.163			0.163		

^a^ *p<0*.*05*

Variable values: Infant feeding pattern (exclusive breastfeeding = 0, predominant breastfeeding = 1, mixed feeding = 2); Number of childbirth (more than one child = 0, one child = 1)

PPWR: postpartum weight retention

## Discussion

### Dietary index for assessment of puerperal women

To our knowledge, this study was the first assessment of overall dietary quality using diet index method for Chinese puerperal women, and other investigations usually analyzed individual nutrients and food groups separately.

The dietary quality depends on the existing dietary patterns, which were assessed generally by two approaches: a priori method based on prior nutrition knowledge translated into dietary guidelines, and posteriori method, in which dietary patterns are extracted once the dietary intake are investigated [9]. In the a priori method, the diet quality indices are constructed to quantify the healthiness of the dietary pattern based on existing scientific guideline [9]. We have finished assessing dietary patterns of Chinese puerperal women in the same cohort (manuscript under submission), and identified four dietary patterns through a posterior principal component analysis, including “plant food” pattern (characterized by high intake of rice and vegetables), “diverse” pattern (characterized by starchy roots, fruit, livestock meat and aquatic products, relatively healthy), “traditional northern” pattern (poultry meat, eggs and soup), and “marine-flour” pattern (flour, coarse food grains and marine foods). In this study the dietary quality of Chinese puerperal women was assessed using a priori approach, i.e. Chinese diet balance index, which has been proved to be an effective index to evaluate overall dietary quality by food adequacy and moderation, developed based on Chinese Dietary Guideline and the Chinese Food Pagoda 2016.

The general DBI-2016 was developed for normal adults and in the study which must be revised to meet the need of puerperal women. One important revision is to delete some DBI components like alcoholic beverages, which consumption was seldom in Chinese puerperal women. In our whole cohort population, the drinking percentage during puerperium was only 1.3% (n = 7), which has been deleted from the study subjects. Similarly, a study conducted by Fernandez-Barres et al. created a new tool, “the relative MD score”, excluding alcohol from the scoring system [32]. We also adjusted other items, including animal foods and dairy products, which are rich in good protein and vitamin A, recommended a higher intake during the lactating period. Population with different physical activity levels require different energy, our study focused on puerperal women whose physical activity is usually defined as low level, and energy requirement is 2300kcal/d [33]. But in the general DBI, the energy levels include 2200Kcal and 2400Kcal without 2300Kcal. Considering the Chinese puerperal women are prescribed animal foods during “doing the month” period, which may exceed the energy recommendation [34], our study estimates 2400Kcal/d as energy level.

Considering the availability of foods and cultural dietary preferences, it is necessary to develop recognized diet indices based on national dietary recommendations, to evaluate the dietary quality of puerperal women for in-country comparison and trend analysis. However, such indices should be used carefully for cross-country comparisons because their generalizability might be limited, especially regarding the foods or food groups included in the index.

### Dietary quality of puerperal women

The results showed the postpartum diet quality was unreasonable and imbalanced, 84.8% of puerperal women had an insufficient dietary intake (especially fruits, vegetables, dairy, beans and aquatic foods), 67.1% had an excessive intake (especially meat and eggs), and 98.4% had an imbalanced diet consumption, that was similar to a posterior analysis. The overall dietary pattern in a prior approach was like “traditional northern” pattern in a posterior approach. These findings are also consistent with other studies in Chinese population. Li et al reported that the puerperal lactating women in Fuzhou had an excessive intake of poultry and eggs, and inadequate intake of vegetables, fruits, milk, and bean products generally [35]. Mao et al found that postpartum women in the central district of China had abundant consumption of cereals and animal foods; however, their intake of dairy products, fruits, and vegetables failed to meet established recommendations [36]. The Chinese National Nutrition and Health Surveillance (2010–2013) showed that during the first month postpartum the percentage of fruits and dairy consumption at least once a day were 67.4% and 38.3%, the median intake of vegetables, fruits and dairy products were 150.0, 40.0 and 0.0 g/d, respectively, much less than recommendation of Chinese Dietary Guideline [37]. Similar results were shown in other countries. A study reported that Spanish lactating women did not meet the recommendations for cereals, salad vegetables, fresh fruits and dairy [38]. Another showed that in North America only 13% and 21% of lactating women had adequate fruit and vegetables respectively, and 9% had a good quality diet [23]. Yu et al. applied dietary modelling method to develop individualized optimal diets, which meet the nutrient requirements for lactating women in urban China, they found that the large difference between observed and optimized diets suggests that the nutrient needs of lactating mothers in China may only be met after substantial dietary changes [39]. Vega-López S et al. using Health Eating Index 2010 assessed dietary quality of Mexican-American postpartum women, all participants in the study had HEI-2010 scores suggesting intake of diets with low quality or in need for improvement (55 ± 14 relative to a maximum score of 100) [40]. Above results indicated the dietary quality of puerperal women was imbalanced in different countries, despite the targeted foods were a little different.

### Dietary quality and PPWR

Gestational weight gain and postpartum weight retention could increase maternal noncommunicable diseases risk and subsequent pregnancy, and the dietary quality was the main influencing factors. Our results showed the percentage of PPWR (≥5kg) were 63.0% at 0–3 months and 52.8% at 6–8 months respectively. Considering dietary patterns present a more holistic view of the overall diet quality, which are currently on the frontier of the association examination between diet and chronic diseases risk [24].

Many other studies demonstrated the relations of energy intake with weight [19, 24], but few studies confirmed the association between postnatal diet and PPWR [25]. Our study did not find an association between overall HBS, LBS, or DQD and PPWR, but revealed a negative correlation between the fish intake score and PPWR at 6–8 months, which indicated that fish intake tended to be a favourable factor for postpartum weight loss. López-Olmedo et al also found that postpartum weight changes were not related to the postpartum diet [25]. The American Infant Feeding Practices Study II showed that despite the apparent difference in the trajectory of weight change by diet quality as assessed by aMED or AHEI-2010 in the early part of the postpartum period, neither total mean weight retention nor the probability of substantial weight retention differed through the end of follow-up, instead, the total energy intake was a significant contributor of substantial weight retention [19]. Donne et al. conducted a study found that small oily fish consumption played a protective role in preterm birth risk, neonatal weight, length and head circumference [26]. Our study found the more fish and shrimp consumption, the lower PPWR at 6–8 postpartum. Agree with above, adequate intake of fish and shrimp is beneficial for pregnant women, postpartum women as well as newborns. Different from our results, a study, included 305 lactating women in south-central China, showed that a diet characterized by high intake of fresh vegetables, soy milk, and bacteria and algae was negatively associated with postpartum weight retention [27]. To date, studies on fish intake and postpartum weight retention are rare and need to be further explored.

Other study found that the postpartum women in higher socioeconomic status (SES) retained less weight [41], the higher SES might mean the higher health literacy of puerperal women and family members, more availability of healthy food, and much more professional care. But our study found that family economic status was not associated with PPWR, the reason perhaps is that the respondents in this study have a higher level of education, with 60.9% having a bachelor’s degree or above. Further studies are needed to provide more evidence for the conclusion.

Our study has certain limitations. We focused on food intake, but not nutrient intake. Despite these limitations, developing DBI-L can not only quantitatively evaluate the dietary quality of lactating mothers, but also have great significance for further exploring the diet of lactating mothers and postpartum weight retention.

## Conclusion

Overall, the dietary quality of puerperal women is an important factor influencing short-term and long-term maternal health and offspring development, it is important to support and engage women throughout this life-stage. To our knowledge, it is the first study to assess the overall dietary quality of Chinese puerperal women using diet index, and the results showed the puerperal women had an imbalanced diet, and fish intake tended to be a favourable factor for postpartum weight loss. Also, many things remained regretted and unclear, the validity and reliability of adjusted DBI should be evaluated, the association of maternal dietary quality with breast milk secretion and infants’ development need to be explored, nutritional intervention should be designed, and so on. Furthermore, special attention should be given to these gaps.

## Supporting information

S1 File (DOCX)

S2 File (DOCX)

S1 Data (SAV)
