# Supplementary material for: Adjusting DBI-2016 to dietary balance index for Chinese maternal women and assessing the association between maternal dietary quality and postpartum weight retention: A longitudinal study
Source: PLoS One. 2020 Aug 20;15(8):e0237225. doi: 10.1371/journal.pone.0237225 (PMC7444517; doi:10.1371/journal.pone.0237225)
Supplement: S1 File — (DOCX) [file pone.0237225.s001.docx]

| **母亲一般情况调查表** | | | |
| --- | --- | --- | --- |
| 监测点： | | | 问卷编码： |
| 产后0-3月调查日期： | | | 产后0-3月调查员： |
| 产后6-8月随访日期： | | | 产后6-8月随访员： |
| 母亲姓名： | | | 电话号码： |
| 邮箱地址： | | | 备用电话号码： |
| 家庭地址： | | | |
| 填写说明： | | （1）本问卷由孩子母亲填写 | |
|  |  | （2）请在横线上填写相应内容，或在相应选项前打“√” | |
|  | 您的年龄 周岁？ | | |
|  | 您的文化程度？ | | |
|  | ①高中及以下 ②本科及以上 | | |
|  | 过去一年，每月人均收入多少元？（包括实物和房屋出租收入等） | | |
|  | ①低于3000元 ②3001-6000元 ③大于6001元 | | |
|  | 本次怀孕期间，您是否出现过下列症状或疾病？（可多选） | | |
|  | ①小腿痉挛 ②牙龈出血 ③妊娠糖尿病 ④妊娠高血压综合症 ⑤贫血 ⑥无 | | |
|  | 您是否有产后大出血？ | | |
|  | ①否 ②是 | | |
|  | 您此次生产后是否发生了产褥感染？ | | |
|  | ①否 ②是 | | |
|  | 您此次生产是否是初产？ | | |
|  | ①否 ②是 | | |
|  | 您的孩子是您怀孕 周生下来的？ | | |
|  | 您此次生产采用哪种分娩方式？ | | |
|  | ①阴道分娩 ②剖腹产 | | |
|  | 您的孩子的出生体重是 千克（1斤=500克=0.5千克）？ | | |
|  | 您目前的喂养方式是？ | | |
|  | ①纯母乳喂养 ②主要母乳喂养 ③混合喂养 ④其他 | | |
|  | 您目前的身高是 厘米（cm）？ | | |
|  | 您本次怀孕前体重 千克（Kg）？ | | |
|  | 您本次临产前体重 千克（Kg）？ | | |
|  | 您目前的体重是 千克（Kg）（产后0-3月） | | |
|  | 您目前的体重是 千克（Kg）（产后6-8月） | | |
|  | 您现在是否饮酒？ | | |
|  | ①否 ②偶尔 ③经常 | | |

| **膳食调查** | | | | | | |
| --- | --- | --- | --- | --- | --- | --- |
| 请回忆在您月子期间里，您是否吃过以下食物，并估计这些食物的平均食用次数和平均每次食用量。  （常用单位换算：1两=50克） | | | | | | |
| 食物名称 | | 选②则跳至问下一类食物 | 进食次数  （以下任选一栏填写） | | | 每次食用量 |
|  |  |  | 次/天 | 次/周 | 次/月 |  |
| 1 | 米及其制品（如米饭、米粉、粥等，按可食部生重计） | ①是 ②否 |  |  |  | 克 |
| 2 | 面及其制品（如馒头、面条等，按可食部分生重计） | ①是 ②否 |  |  |  | 克 |
| 3 | 粗粮（如玉米制品、大麦、小米、荞麦等，按可食部分生重计） | ①是 ②否 |  |  |  | 克 |
| 4 | 薯类（如白薯、山药、芋头等，按可食部分生重计） | ①是 ②否 |  |  |  | 克 |
| 5 | 深色蔬菜（如菠菜、胡萝卜、西红柿等，按可食部分生重计） | ①是 ②否 |  |  |  | 克 |
| 6 | 浅色蔬菜（如白菜、黄瓜等，按可食部分生重计） | ①是 ②否 |  |  |  | 克 |
| 7 | 水果（按可食部分生重计） | ①是 ②否 |  |  |  | 克 |
| 8 | 畜肉（如牛肉、猪肉、羊肉、动物肝脏等，按可食部分生重计） | ①是 ②否 |  |  |  | 克 |
| 9 | 禽肉(如鸡肉、鸭肉等) | ①是 ②否 |  |  |  | 克 |
| 10 | 除海鱼外的其他水产品（按可食部分生重计） | ①是 ②否 |  |  |  | 克 |
| 11 | 海鱼（按可食部分生重计） | ①是 ②否 |  |  |  | 克 |
| 12 | 蛋类（鸡蛋、鸭蛋、鹌鹑蛋等） | ①是 ②否 |  |  |  | 克 |
| 13 | 奶及奶制品（牛奶、奶粉等） | ①是 ②否 |  |  |  | 克 |
| 14 | 豆浆 | ①是 ②否 |  |  |  | 毫升 |
| 15 | 豆类及其制品（如大豆、黄豆、豆腐  /豆干等，按可食部分生重计） | ①是 ②否 |  |  |  | 克 |
| 16 | 汤水（如鸡汤、骨头汤等） | ①是 ②否 |  |  |  | 毫升 |
| 17 | 盐 | ①是 ②否 |  |  |  | 克 |
| 18 | 油脂类（植物油、动物油等） | ①是 ②否 |  |  |  | 克 |
| 19 | 饮料类（碳酸饮料、果蔬饮料、奶茶等） | ①是 ②否 |  |  |  | 毫升 |
| 20 | 零食类（方便面、饼干、糕点、糖果、巧克力、果冻、薯片、牛肉干、火腿肠、坚果类等） | ①是 ②否 |  |  |  | 克 |
| 21 | 营养素补充剂（钙、铁、锌、叶酸、维生素A、维生素D、复合维生素B、  DHA/鱼肝油等） | ①是 ②否 |  |  |  | —— |
