# Supplementary material for: Adjusting DBI-2016 to dietary balance index for Chinese maternal women and assessing the association between maternal dietary quality and postpartum weight retention: A longitudinal study
Source: PLoS One. 2020 Aug 20;15(8):e0237225. doi: 10.1371/journal.pone.0237225 (PMC7444517; doi:10.1371/journal.pone.0237225)
Supplement: S2 File — (DOCX) [file pone.0237225.s002.docx]

| **Part 1** | | | |
| --- | --- | --- | --- |
| Location： | | | ID： |
| Date(0-3 mouth postpartum)： | | | Investigator(0-3 mouth postpartum)： |
| Date(6-8 mouth postpartum)： | | | Investigator(6-8 mouth postpartum)： |
| Name： | | | Tel： |
| Email： | | | Tel: |
| Address： | | | |
| Directions | | （1）Read each question carefully | |
|  |  | （2）Draw“√”on your answer sheet that match your answer | |
|  | Age: ？ | | |
|  | The highest level of education you have completed is ？ | | |
|  | ①High school or below ②Bachelor degree or above | | |
|  | Household monthly income per capita (CNY) | | |
|  | ①Less than ￥3000 ②￥3001-￥6000 ③More than ￥6001 | | |
|  | Did you experience any of the following symptoms or illnesses during pregnancy? (Single or Multiple choice) | | |
|  | ① Calf spasm ② Gingival bleeding ③ Gestational diabetes ④ Gestational hypertension syndrome ⑤ Anemia ⑥ None | | |
|  | Did you experience major postpartum hemorrhage? | | |
|  | ①No ②Yes | | |
|  | Did you have a puerperal infection after this birth? | | |
|  | ①No ②Yes | | |
|  | If this is your first birth? | | |
|  | ①No ②Yes | | |
|  | Gestational age？ | | |
|  | ①Full term infant ②preterm infant ③post-term infant | | |
|  | Delivery method？ | | |
|  | ① Vaginal delivery ② Caesarean delivery | | |
|  | Infant birth weight Kg？ | | |
|  | Feeding method？ | | |
|  | ①Exclusive breastfeeding ②Predominant breastfeeding ③Mixed feeding ④Neither | | |
|  | Current Hight: cm？ | | |
|  | Pre-pregnancy weight: Kg？ | | |
|  | Weight at the time of delivery: Kg？ | | |
|  | Weight at 0-3 mouth postpartum Kg? | | |
|  | Weight at 6-8mouth postpartum Kg? | | |
|  | If you drink alcohol now？ | | |
|  | ①No ②Occasionally ③Often | | |

| **Part 2** | | | | | | |
| --- | --- | --- | --- | --- | --- | --- |
| Please recall if you have eaten the following foods in the past month, and estimate the frequency and average intake of these foods. (1 Liang = 50g). Dietary intake based on the edible weight. | | | | | | |
| Food items | | If you choose ②, skip to the next question | Frequency of dietary intake | | | quantity |
|  |  |  | Per day | Per week | Per month |  |
|  | Rice and its products (such as rice, rice flour, porridge, etc.) | ①Y ②N |  |  |  | g |
|  | Wheat and its products (such as wheat bun, wheat noodles, what pancake, etc.) | ①Y ②N |  |  |  | g |
|  | Coarse grains (such as corn products, barley, millet, buckwheat, etc.) | ①Y ②N |  |  |  | g |
|  | Potatoes (such as sweet potatoes, yam, taro, etc.) | ①Y ②N |  |  |  | g |
|  | Dark vegetables (such as spinach, carrots, tomatoes, etc.) | ①Y ②N |  |  |  | g |
|  | Light-colored vegetables (eg cabbage, cucumber, etc.) | ①Y ②N |  |  |  | g |
|  | Fruit | ①Y ②N |  |  |  | g |
|  | Red meat and products (such as beef, pork, mutton, animal liver, etc.) | ①Y ②N |  |  |  | g |
|  | Poultry and game (e.g. chicken, duck, etc.) | ①Y ②N |  |  |  | g |
|  | Other aquatic products other than marine fish | ①Y ②N |  |  |  | g |
|  | Marine fish | ①Y ②N |  |  |  | g |
|  | Eggs (eggs, duck eggs, quail eggs, etc.) | ①Y ②N |  |  |  | g |
|  | Milk and dairy products (milk, milk powder, etc.) | ①Y ②N |  |  |  | g |
|  | Soy milk | ①Y ②N |  |  |  | ml |
|  | Soybean and products (such as soybeans, soybeans, tofu, Dried tofu, etc.) | ①Y ②N |  |  |  | g |
|  | Soup (such as chicken soup, bone soup, etc.) | ①Y ②N |  |  |  | ml |
|  | Salt | ①Y ②N |  |  |  | g |
|  | Oils and fats (vegetable oil, animal oil, etc.) | ①Y ②N |  |  |  | g |
|  | Beverages (carbonated drinks, fruit and vegetable drinks, milk tea, etc.) | ①Y ②N |  |  |  | ml |
|  | Snacks (instant noodles, biscuits, pastries, candy, chocolate, jelly, potato chips, beef jerky, ham sausage, nuts, etc.) | ①Y ②N |  |  |  | g |
|  | Nutrient supplements (calcium, iron, zinc, folic acid, vitamin A, vitamin D, multivitamin B, DHA / cod liver oil, etc.) | ①Y ②N |  |  |  | —— |
